# The Neural Correlates of Long-Term Carryover following Functional Electrical Stimulation for Stroke

**DOI:** 10.1155/2016/4192718

**Published:** 2016-03-17

**Authors:** Marta Gandolla, Nick S. Ward, Franco Molteni, Eleonora Guanziroli, Giancarlo Ferrigno, Alessandra Pedrocchi

**Affiliations:** ^1^Department of Electronics, Information and Bioengineering, Polytechnic University of Milan, Via G. Colombo 40, 20133 Milan, Italy; ^2^UCLP Centre for Neurorehabilitation, Queen Square, London WC1N 3BG, UK; ^3^Sobell Department of Motor Neuroscience, UCL Institute of Neurology, Queen Square, London WC1N 3BG, UK; ^4^The National Hospital for Neurology and Neurosurgery, Queen Square, London WC1N 3BG, UK; ^5^Valduce Hospital, Villa Beretta Rehabilitation Center, Via N. Sauro 17, 23845 Costa Masnaga, Italy

## Abstract

Neurorehabilitation effective delivery for stroke is likely to be improved by establishing a mechanistic understanding of how to enhance adaptive plasticity. Functional electrical stimulation is effective at reducing poststroke foot drop; in some patients, the effect persists after therapy has finished with an unknown mechanism. We used fMRI to examine neural correlates of functional electrical stimulation key elements, volitional intent to move and concurrent stimulation, in a group of chronic stroke patients receiving functional electrical stimulation for foot-drop correction. Patients exhibited task-related activation in a complex network, sharing bilateral sensorimotor and supplementary motor activation with age-matched controls. We observed consistent separation of patients with and without carryover effect on the basis of brain responses. Patients who experienced the carryover effect had responses in supplementary motor area that correspond to healthy controls; the interaction between experimental factors in contralateral angular gyrus was seen only in those without carryover. We suggest that the functional electrical stimulation carryover mechanism of action is based on movement prediction and sense of agency/body ownership—the ability of a patient to plan the movement and to perceive the stimulation as a part of his/her own control loop is important for carryover effect to take place.

## 1. Introduction

Stroke is one of the leading causes of adult disability [1]. Advances in acute treatment have led to improvements in survival and so establishing effective rehabilitation strategies has become even more important. This process is likely to be facilitated by establishing a mechanistic understanding of how to use adjunctive therapies to augment standard rehabilitation approaches.

Functional electrical stimulation (FES) is a commonly used adjunctive therapy in the rehabilitation of stroke [2]. It is primarily used for the orthotic correction of foot drop, but a proportion of patients relearn the ability to voluntarily dorsiflex the ankle without the device [3]. This phenomenon, referred to as the “carryover effect,” has been observed in a number of subsequent studies [4, 5]. To date, the mechanism of this effect is unknown, although it has been hypothesised that an interaction between volitional effort and the electrical stimulation of FES results in a neuroplastic effect on the central nervous system [6–9]. However, the carryover effect has been observed only in subgroups of neurological patients and the characteristics of those with and without FES carryover are not clear.

In this work, we used functional magnetic resonance imaging (fMRI) to examine the neural correlates of the key ingredients of FES, namely, volitional intent to move and concurrent electrical stimulation, in a group of chronic stroke patients receiving FES treatment for foot drop. We were then interested to see whether brain activity during movement or stimulation (or the interaction between the two) before FES treatment would have any value in predicting whether individual patients would have a carryover effect on volitional ankle dorsiflexion after removal of FES.

In healthy subjects, the interaction between volitional movement and proprioceptive feedback occurs in primary sensory and motor cortex [9]. We might expect to see “normal” brain response to the elements of FES in those with carryover and diminished responses in those without carryover. However, sensorimotor systems are organised differently after stroke particularly in primary and secondary areas. The interaction (or lack of it) between volitional movement and proprioceptive feedback might therefore occur in nonprimary sensory (secondary somatosensory area, SII, and posterior parietal cortex) and motor (lateral and medial premotor cortex) areas. The sensitivity and spatial resolution of fMRI allow us to make specific conclusions about the cortical regions active during key elements of FES and to investigate the likely neural mechanism of the carryover effect following FES treatment.

## 2. Materials and Methods

### 2.1. Participants

Patients were recruited from the outpatient and inpatient services at the Villa Beretta Rehabilitation Centre (Costa Masnaga, LC, Italy). All patients had suffered from first-ever stroke > 6 months previously, resulting in weakness of at least the tibialis anterior muscle (to <4+ on the Medical Research Council (MRC) scale [10]). Exclusion criteria consisted of (i) less than 10° in FES-induced ankle dorsiflexion; (ii) language or cognitive deficits sufficient to impair cooperation in the study; (iii) inability to walk even if assisted; (iv) high spasticity at ankle joint plantar flexor as measured by the modified Ashworth scale index > 2 [11].

The age-matched control group was composed of healthy volunteers with no neurological or orthopaedic impairment. Their results have been fully reported previously [9].

Experiments were conducted with approval from the Villa Beretta Rehabilitation Centre Ethics Committee and all subjects gave informed written consent in accordance with the Declaration of Helsinki.

### 2.2. Clinical and Instrumental Measures for Impairment Assessment

Patients' impairment at the time of recruitment for this study was evaluated using a battery of clinical and instrumental tests. In particular, they were evaluated through a gait analysis test performed following the standard Davis evaluation protocol [12] along with the correspondent dynamic electromyography test and the 6-minute walking test [13]. Moreover, they were scored by the clinician on the MRC scale index at ankle dorsiflexion.

From these tests, a set of five outcome measures was designed to assess different aspects of patients' functional condition:

- Gait velocity as measured during the gait analysis test.
- Endurance velocity, as calculated during the 6-minute walking test.
- Paretic step length as measured during the gait analysis test.
- Tibialis Anterior Activation Index defined as the ratio between the activity of the tibialis anterior muscle between toe off and toe strike and that during the whole gait cycle [14].
- MRC index [10].

Patients were trained 5 times per week for 4 weeks. Each of these 20 sessions consisted of 30 minutes of walking supported by a commercial electrical stimulator. Two commercial devices were available at the Villa Beretta Rehabilitation Centre: Bioness L300 (Bioness Inc.) and WalkAid (Innovative Neurotronics). Two stimulating electrodes were placed superficially along the peroneal nerve to elicit tibialis anterior muscle contraction during the swing phase of gait. Swing phase was detected online by wireless heel switches (Bioness) or by accelerometers (WalkAid). The more suitable commercial device was selected for each patient depending on his/her best responsiveness to stimulation, best wearability, and reliability of swing phase detection. Current stimulation amplitude was selected for each participant at the beginning of each session so as to be able to elicit ankle dorsiflexion during gait but at the same time to remain within the tolerance level.

Impairment was evaluated at the time of recruitment for this study (*t*_1_: within 5 days before the start of the treatment) and after the intervention (*t*_2_: within 5 days since the end of the treatment) using the same battery of clinical and instrumental tests.

The carryover effect was determined using a novel algorithm based on variables minimum detectable change that combines the outcome measures to obtain a unique parameter, Capacity Score, where a higher Capacity Score indicates higher residual ability. In particular, for each assessment session, patients were assigned to a point in an *n*-dimensional space, where *n* corresponds to the number of outcome measures considered. The *n*-dimensional space was centred on the outcome measures derived from healthy subjects, and therefore the further away the patient is from the origin, the more impaired he/she is. Moreover, the outcome variables have been normalized with respect to the corresponding minimum detectable change. The difference in the *n*-dimensional space between (i) the Euclidean distance of “subject zero” (a patient that scores zero in all outcome measures, i.e., the most impaired patient in our space) with respect to the origin (i.e., distance of the “subject zero” from the healthy control group) and (ii) the Euclidean distance of each patient with respect to the origin (i.e., distance of the “subject zero” from the healthy control group) is defined as Capability Score. The difference between Capacity Scores at different timing (i.e., post-pre) is thresholded to obtain carryover effect assessment. The algorithm has been validated against clinical evaluation [15].

### 2.3. Experimental Setup

The experimental setup was composed of 1.5 T MRI scanner (GE Cv/I), a motion capture system (Smart *μ*g; BTS), and an electrical stimulator (RehaStim proTM; HASOMED GmbH), as previously described and validated [16, 17].

### 2.4. Experimental Design

A 2 × 2 event-related fMRI design was carried out. Experimental factors were (i) volitional intention to perform ankle dorsal-plantar flexion (ADF) [V: with the levels “volitional” and “passive”] and (ii) FES [F: with the levels “present” and “absent”]. Each patient was instructed to execute the protocol with the plegic ankle. During a continuous 10-minute scanning session, subjects performed 20 alternate 9-second OFF and 21-second ON blocks. The 4 conditions that constituted our factorial design were performed during the ON blocks in a semirandomized order: (i) FV: attempted voluntary ADF with concurrent FES-induced ADF; (ii) FP: FES-induced ADF, with no attempt to move the ankle; (iii) V: voluntary ADF without FES; (iv) P: passive dorsiflexion (by the experimenter) of the subject's ankle without FES. Subjects were specifically instructed to remain completely relaxed during FP and P conditions and to equally voluntarily contribute during V and FV conditions. Dorsiflexion was paced every 3.5 seconds (for 6 repetitions within a block) with an auditory cue (i.e., movement rate 0.3 Hz) [18]. The auditory cues were presented through an earphone. Prior to scanning, subjects practiced the protocol until being comfortable with the task; the experimenter was assisting the training to check the correct execution of the protocol and equate effort across subjects. All subjects were free to choose the amplitude of their active movement to preclude fatigue. Indeed, Ciccarelli and colleagues [18] did not find any effect of movement amplitude (10°–55°) on magnitude or pattern of brain activity, suggesting that if the movement does not have any external reference and it is self-paced, there is no difference due to amplitude in associated cortical activity. Subjects were instructed to keep eyes closed and head movements were minimized with rubber pads and straps. To ensure minimum transmission of movements to the head, knees were bent with the subject's legs lying on a pillow.

### 2.5. FES Stimulation Paradigm

Functional electrical stimulation was applied to the peroneal nerve through superficial self-adhesive electrodes, with biphasic balanced current pulses at 20 Hz fixed frequency. The pulse width had a trapezoidal profile (maximum pulse width 400 *μ*s) and the current amplitude was set subject by subject so as to produce ADF movement, within the tolerance threshold. Current amplitude and pulse width were kept the same for both FP and FV conditions.

### 2.6. Images Data Acquisition

A GE Cv/I system, operating at 1.5 T, was used to acquire both T1-weighted anatomical images (0.94 × 0.94 × 4 mm voxels) and T2^*∗*^-weighted MRI transverse echo-planar images (1.8 × 1.8 × 4 mm voxels, TE = 50 ms) with blood oxygenation level dependent contrast. Each echo-planar image comprised 22 contiguous axial slices, positioned to cover the temporoparietal and occipital lobes, with an effective repetition time of 3 seconds per volume. Due to technical reasons, it was not possible to acquire the cerebellum. The first six volumes were discarded to allow for T1 equilibration effects. A total of 200 brain volumes were acquired in a single run lasting 10 minutes.

### 2.7. Kinematic Measures and Analysis

A motion capture system previously validated for recording during scanning allowed us to record 3D trajectories of retroreflective markers to measure the ankle angle during fMRI acquisitions and to determine the movement onset for event-related fMRI time series analysis. Two separate acquisition sessions were performed. The first was a static acquisition performed before the scanning, but while lying in the scanner, to estimate the coordinates of the medial and lateral malleoli for both lower limbs. During the static acquisition, a plate with 3 markers was placed on each tibia and 4 sticks with two markers each were placed on the four malleoli. The relative positions of the malleoli with respect to the plates (i.e., left and right plates) were computed and the transformation matrices were estimated under the assumption that tibia and malleoli were rigidly connected. The second acquisition, dynamic acquisition, was performed during the fMRI scanning. Only the two plates on the tibia were used to estimate the tibia 3D position and the malleoli. Four additional markers were placed over the four metacarpi. Markers were always visible during ADF for all different conditions. The sampling frequency was set at 120 Hz.

Markers trajectories were analysed with a custom algorithm running in Matlab (MatlabR2010b) to obtain onsets and amplitude of ADF movements. In particular, for each leg, the ADF angle was calculated as follows: the mean points between the medial and lateral malleoli (mean malleolus) and between the medial and lateral metacarpi (mean metacarpus) were calculated. The ADF angle was taken as the angle between the line passing through the more proximal tibial marker and the mean malleolus and the line passing through the mean malleolus and the mean metacarpus [9].

### 2.8. fMRI Data Preprocessing

Imaging data were analysed using Statistical Parametric Mapping (SPM8, Wellcome Department of Imaging Neuroscience, http://www.fil.ion.ucl.ac.uk/spm/) implemented in Matlab (MatlabR2010b). A skull stripping procedure, on the structural image for each subject, was performed to improve the coregistration of functional and structural images. Participants with right-sided infarcts (left-leg weakness) were flipped about the midsagittal line, such that all subjects were considered to have left-sided infarcts. All fMRI volumes were then realigned and unwarped to suppress task-related motion artefacts [19]. Realignment parameters were assessed for excessive motion after unwarping procedure. A threshold of 4 mm in translation and 5° in rotation was applied [20]. The skull stripped structural image was then coregistered to the mean image of the functional realigned volumes and segmented. The spatial normalization transformation (to the Montreal Neurological Institute (MNI) reference brain in Talairach space [21]) was then estimated using the segmented structural image. The structural image and functional volumes were normalized and resampled to 2 mm × 2 mm × 2 mm voxels. Functional normalized images were then smoothed with an isotropic 8 mm full-width half-maximum kernel [22]. The time series in each voxel were high pass filtered at (1/128) Hz during subsequent modelling to remove low frequency confounding factors.

### 2.9. Statistical Analysis

Statistical analysis was performed in two stages using the standard summary statistic approach. In the first stage, functional images were analysed separately for each patient. We were interested in the analysis of brain regions active during each condition, (i) FV, (ii) V, (iii) FP, and (iv) P, as well as (v) their interaction, defined as (FV-V) versus (FP-P) which identifies regions in which the effect of FES is modulated by the presence or absence of volitional intent. From the kinematic measures, two ADF covariates were defined for each condition: onsets and amplitude covariates. All ADF onsets belonging to the same condition were defined as a single event type and modelled as delta functions in the corresponding stimulus function. The amplitude covariate was defined as a delta function scaled by the actual amplitude of each ADF for each condition, and it was mean corrected and orthogonalised with respect to the corresponding onset covariate [23]. All onset and amplitude stimulus functions were then convolved with a canonical hemodynamic response function, together with its temporal and dispersion derivatives [23], and used as regressors in a general linear model of the observed fMRI time series. Linear contrasts of parameter estimates were generated for each subject (i.e., contrast images) and used for the creation of statistical parametric maps at the second (between-subject) stage.

In the second stage, the following analyses were performed:

(i) One-sample *t*-tests were performed using appropriate contrast images for each condition to investigate the main effects of each condition in the stroke group.
(ii) Two-sample *t*-tests using appropriate contrast images for each subject were performed to examine the differences between patients and control subjects groups for each condition. Conjunction analyses (i.e., two separate null hypotheses to be contemporarily denied) were performed between groups for each condition to determine common activation.
(iii) Contrast images were entered into a regression analysis with subject-specific values for the carryover effect to investigate whether there are any areas in which pretreatment brain activity (in any of the conditions) correlates with the subsequent carryover effect of each patient.

Results of all second-stage analyses were thresholded at *p* < 0.05 corrected for multiple comparisons within specific regions of interest (ROIs). Our predefined area of investigation included the following seven areas bilaterally: leg primary motor (M1) and sensory (S1) cortices, secondary somatosensory area (SII), parietal rostroventral area (PR), supplementary motor area (SMA), premotor cortex (PM), and angular gyrus (AG). In particular, our predefined area of investigation included the following seven areas bilaterally: leg primary motor (M1) and sensory (S1) cortices, secondary somatosensory area (SII), parietal rostroventral area (PR), supplementary motor area (SMA), premotor cortex (PM), and angular gyrus (AG). Contralateral primary sensorimotor cortex (i.e., M1, S1) is the primary site of ADF control, and it has been shown to be the site of interaction between volitional intention and FES in healthy controls [9]. In turn, ipsilateral primary sensorimotor cortex has been demonstrated to be active for poststroke patients while executing simple motor tasks [24]. The two secondary somatosensory areas (cSII, i cSII) have been selectively linked to proprioceptive processing and integration [25], attention to proprioceptive stimuli [26], painful and nonpainful stimulus processing [27], and complex object manipulation [28]. Moreover, the secondary somatosensory area has been demonstrated to be active during stimulation nonselectively [9] and selectively [7] with respect to voluntary effort in controls. PR areas have been identified as potential sites of sensorimotor integration [25] by virtue of their anatomical connections with premotor and primary motor cortices [29]. PR area has been shown to have an activation pattern similar to SII and so might have a similar role in processing sensorial stimuli [9, 25]. Bilateral premotor and supplementary motor areas are a highly consistent finding after stroke during simple motor tasks execution [30]. Right AG has been demonstrated to be the site of self-representation of movement [7, 31], and it has been demonstrated to be more active during passive than active FES. Moreover, AG has been suggested to be the recipient of proprioceptive information encoded in the postcentral gyrus [32].

Based on previous work, ROIs were defined as follows using the MNI coordinates system. Primary motor (M1) and primary sensory cortices (S1) were defined as 10 mm spheres centred, respectively, on [*x* = ±6, *y* = −28, *z* = 60] and [*x* = ±4, *y* = −46, *z* = 62] [33]; bilateral secondary somatosensory cortices (SII) as 10 mm spheres centred on [*x* = ±58; *y* = −27; *z* = 30] [7, 34]; PR as 10 mm spheres centred on [*x* = ±54; *y* = −13; *z* = 19] [25]; SMA and PM were defined as 15 mm spheres centered, respectively, on [±20; −8; 64] and [±8; −6; 64] [34]. AG was anatomically defined using the WFU PickAtlas [35]. Anatomical attribution was performed by carefully superimposing the maxima of significant effects both on the MNI brain and on the normalized structural images averaged across all subjects and then labelling with the aid of the atlas of Duvernoy [36].

## 3. Results

### 3.1. Participants

The healthy control group was aged between 22 and 61 years [mean (standard deviation): 36 (14) years], comprising 8 male and 9 female subjects. Fourteen poststroke patients were recruited [range: 19–64 years, mean (standard deviation): 44 (14)], comprising 8 male and 6 female subjects. There was no significant difference between the groups in terms of age (*p* = 0.15). Patient characteristics along with the degree of functional recovery at the time of scanning as measured by the selected outcome measures are listed in Table 1.

**Figure 1:**
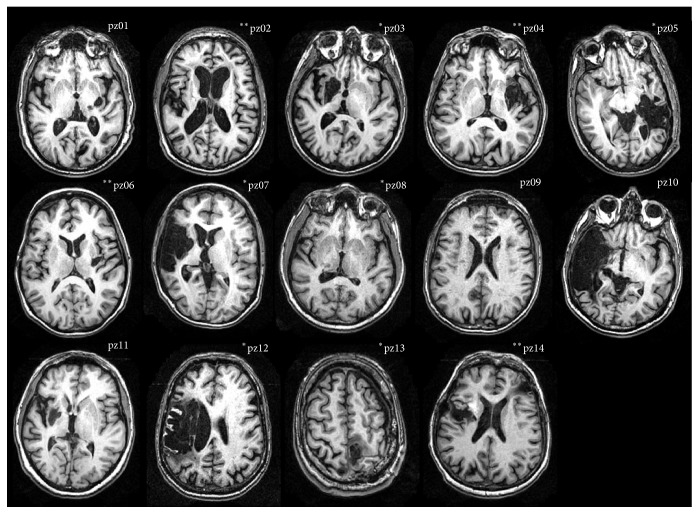
The site of cerebral infarction as determined from the T1-weighted structural MRI. *∗∗*: patient with FES carryover (responder); *∗*: patient with no FES carryover (i.e., nonresponder).

For eight patients, stroke resulted in left hemiparesis and for six in right hemiparesis. The site of cerebral infarction was determined from the T1-weighted structural MRI (Figure 1).

The carryover effect was not predicted by age, sex, side of the lesion (i.e., right/left), time since stroke acute event, the baseline impairment, and the type of device used for training, as determined by multiple linear regression.

Ten out of the fourteen patients completed the FES-based rehabilitation treatment, receiving the same dosage of therapy, and so we were able to assess the carryover effect in only a subgroup of participants (Table 1). A follow-up assessment was planned for all patients one month after the end of the treatment to check for long-term effects of carryover. Six patients were available for this extra visit, and the carryover effect presence/nonpresence was demonstrated to be stable for all patients but for one where the effect was no longer present [15]. The patients that did not complete the treatment/assessment were outpatients, and they discontinued the treatment for personal reasons, mainly linked to difficulties in logistically managing an everyday treatment in clinic or managing a further travel.

### 3.2. Kinematic Measures

Mean ADF amplitude across subjects along with its standard deviation for V condition was 18° ± 11°, for FV condition 20° ± 11°, for FP condition 18° ± 11°, and for P condition 21° ± 13°. All ADF angles for all subjects were within the 10°–70° interval [18].

### 3.3. Images Analysis

All controls and patients were able to perform the task adequately. No subjects displayed mirror movements at bedside observation or when performing the motor paradigm outside the scanner. However, a number of patients did exhibit synergistic contralateral ankle dorsiflexion during motor paradigm execution in the scanner (Table 1). However, a linear regression analysis with mirror movements (i.e., present/absent) as independent categorical variable and carryover effect as dependent variable demonstrated the independence of the two elements (*p* value = 0.4860).

Realignment parameters were assessed for excessive motion after unwarping procedure, and the maximum translational displacement was 0.27 mm in all directions and the maximum rotational displacement was 0.0029°.

**Figure 2:**
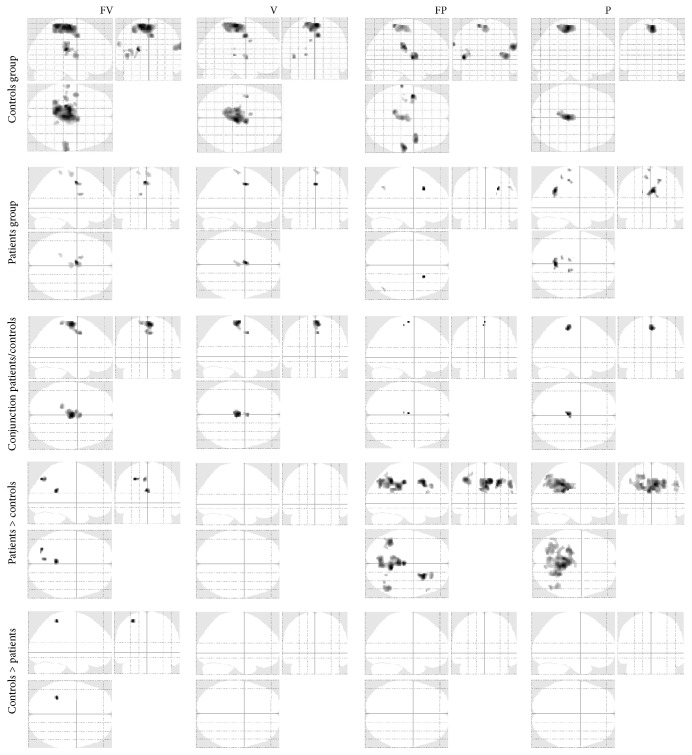
Activation maps for the experimental conditions. Regions active during each experimental condition (i.e., V, FV, FP, and P) in the controls and patients groups separately, as well as the conjunction analysis, and comparison between patients and controls. Statistical parametric maps (thresholded at *p* < 0.001, uncorrected for display purposes) showing regions activated in the four conditions using a maximum intensity projection format.

**Figure 3:**
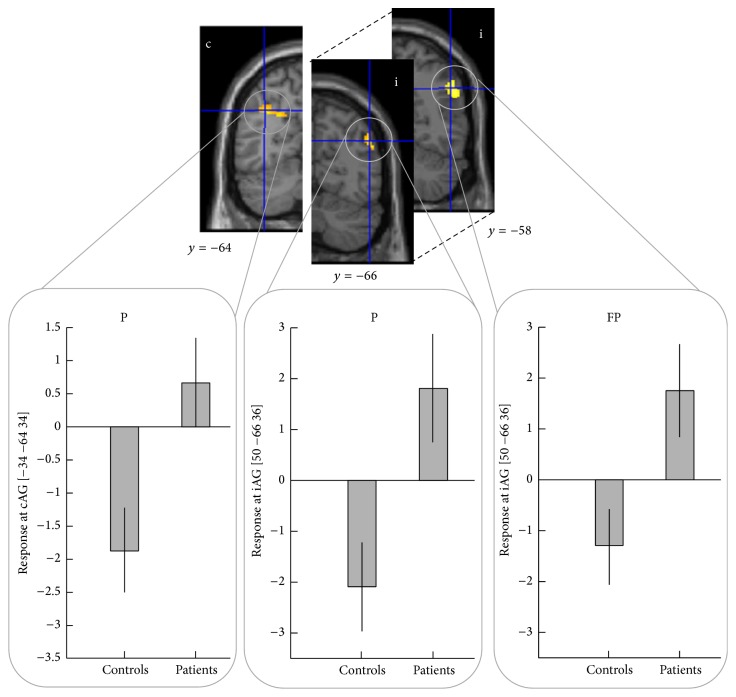
Brain responses in controls and patients groups in angular gyrus (AG). P: passive experimental condition; FP: FES passive experimental condition; c: contralateral; i: ipsilateral.

**Figure 4:**
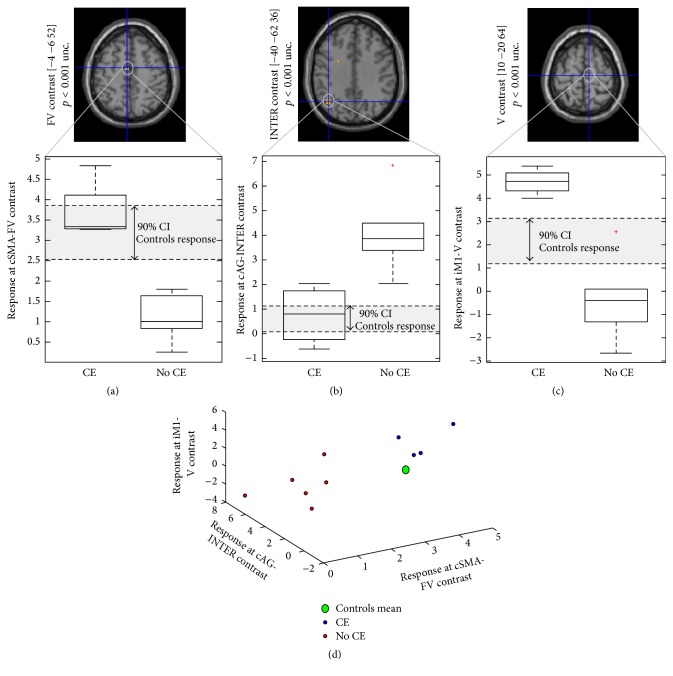
Separation of patients with and without carryover effect on the basis of the brain responses. Response for the peak voxel for cSMA (a), cAG (b), and iM1(c) areas in patients who experienced/did not experience the carryover effect. (d) 3D representation of the responders/nonresponders groups on the basis of brain responses. CE: patients group with FES carryover (responders); non-CE: patients group with no FES carryover (nonresponders); FV: FES with volitional contribution; INTER: interaction contrast; V: volitional ankle dorsiflexion; SMA: supplementary motor cortex; M1: primary motor cortex; AG: angular gyrus; c: contralateral; i: ipsilateral.

Figure 2 shows brain regions active during each condition (i.e., V, FV, FP, and P) in the controls and patients groups separately, as well as the conjunction analysis, and comparison between patients and controls:

- Patient group: as reported in Table 2, patients show task-related activation in primary and secondary sensorimotor areas, in contralateral paracentral lobule [37], bilateral frontal cortex, cingulate gyrus, precuneus, and supramarginal gyrus. AG area is only activated by patients, ipsilaterally in FP condition, and contralaterally shows an interaction between design factors (i.e., volitional intention and FES).
- Patients versus healthy controls: controls show clear activation in all conditions in motor and somatosensory areas known to be involved in ADF execution and in accord with previous studies [7, 9, 18], as expected. Patients and controls primarily show common activation in bilateral sensorimotor (all conditions) and supplementary motor (for conditions where volitional intention is present: FV, V) areas. However, compared to the control group, patients tend to overactivate right AG when there is no volitional intention to move (i.e., FP, P conditions) and left intraparietal sulcus during passive movement (Figure 3).
- Prediction of carryover effect in patient group: in those patients with carryover after FES, contralateral SMA was more active during stimulation and voluntary conditions (FV, V, and FP conditions), and ipsilateral M1 was more active during voluntary movement (V condition). In those without carryover effect, we saw a greater interaction between factors, that is, (FV-V) > (FP-P) in contralateral AG. By looking at the response for the peak voxel for cSMA, iM1, and cAG areas (Figures 4(a)–4(c)), it can be seen that patients who experienced the carryover effect have responses in SMA and M1 that correspond to healthy controls, whilst responses in these regions in patients without carryover are diminished. Conversely, the interaction between factors in contralateral AG is seen only in those without carryover but not in those with carryover or healthy controls. In other words, we see quite consistent separation of those with and without FES carryover on the basis of the brain responses in these regions (Figure 4(d)). In particular, those with FES carryover appear to have “normal” responses, whilst those without do not.

## 4. Discussion

A prolonged carryover effect from FES is a desirable rehabilitation outcome. Knowing who is most likely to achieve this and how would be useful in a stratified rehabilitation strategy. The motivation for this study was therefore to explain how a peripheral stimulus can facilitate long-term motor relearning (i.e., FES carryover) after a lesion in the central nervous system [6, 38]. It has been suggested that the mechanism of FES carryover is central in origin [6], but this has never been tested in neurological patients. In this study, we have used functional brain imaging to examine brain responses to key components of FES (electrical stimulation and attempted volitional movement, both separately and in combination) that characterise those who exhibit the carryover effect. These characteristics might be considered as biomarkers for successful FES-based rehabilitation after stroke.

Our results point to supplementary motor area (SMA) and angular gyrus (AG) as key regions involved in mediating the carryover effect, since they are differentially active during the key components of FES in those patients with and without carryover. It is first worth considering the normal roles of SMA and AG in sensorimotor tasks. SMA is linked to movement preparation and planning and is often noted to be overactive compared to controls during attempted movement in chronic stroke subjects [39]. Indeed, in our patients, SMA is bilaterally active during conditions where volitional intention is present (i.e., FV, V), as expected. In turn, AG appears to be a recipient of proprioceptive information and a specific area for somatosensory calculation of the reach vector during upper limb reaching [32]. AG processes discrepancies between intended action and movement consequences in such a way that these will be consciously detected by the subject. It has been suggested that AG is activated during intersensory conflicts that may result in a loss of body ownership [31, 40]. On the other hand, damage to AG results in altered awareness of voluntary action [41].

Our results show that those with and without FES carryover have opposite patterns of activity in SMA and AG. In particular, those who have FES carryover exhibit SMA activation during concurrent FES and volitional movement (i.e., FV condition), but they do not show an interaction between FES and volitional movement in AG. In both instances, the neural responses to key elements of FES are more like a normal healthy subject, whereas those without carryover have “abnormal” responses in both SMA and AG (Figure 4). We therefore suggest that the carryover effect is mediated through movement prediction (SMA area) and sense of agency/body ownership (AG area). Specifically, the concept of sense of agency appears to be neuroanatomically associated with primary [31] and secondary [42] sensorimotor areas. The prediction of the sensory consequences of a self-generated action is compared against the actual sensory consequences, where stronger correspondence is associated with a stronger experience of agency (i.e., self-generated action). In other words, those with FES carryover correctly plan the movement when executing the movement with concurrent volitional intention and FES, and as a consequence movement is perceived as self-generated. By doing so, the patient correctly updates the motor control loop [43] that likely enhances a long-term potentiation effect following Hebbian principles. Indeed, the combination of volitional effort and the perception of a “normally” completed movement provides somatosensory feedback that facilitates Hebbian-like plasticity [44]. This is in line with the suggestion that gradual motor learning/adaptation might be also mediated by extracerebellar mechanisms [45] and that the generalization of learning (in particular adaptive learning) is improved when the nervous system assigns errors to “self” rather than the environment [46] or, in this case, the device.

In healthy control subjects, the interaction between volitional intent to move and proprioception was mediated by primary motor and somatosensory areas [9]. In our poststroke patients, bilateral primary sensorimotor cortices are active under all conditions but are not differentially activated by our experimental factors. The function of primary sensorimotor cortices role in mediating the FES effect might therefore be supported by secondary areas, representing a plasticity mechanism that exploits available resources [47]. In fact, it has been repeatedly shown that normal input/output processes of primary sensorimotor cortices are impaired in many neurological patients, with consequent recruitment of a complex network that includes primary and secondary areas to generate even simple motor tasks [34, 48].

The other predefined areas of investigation do not appear to be crucial nodes for mediating FES carryover. In particular, although premotor areas are known to be overactive in many stroke patients [46], they do not appear to be crucial for our patients during ADF, being only ipsilaterally active in FV condition. It has been suggested that the human SI and SII cortices may be sequentially activated within one hemisphere, whereas SII ipsilateral to the stimulation may receive direct input from the periphery, at least when normal input from SI is interrupted [49]. In our control group, bilateral SII was active for the FES conditions (i.e., FV, FP), possibly as the direct recipient of the stimulus [8, 50]. In patients, we observed only ipsilateral SII activation, which preserves its presumed role of processing electrical stimulation as in healthy controls [9, 49, 50]. On the other hand, we suggest either that contralateral SII is not primarily involved in somatosensory information processing or that contralateral stimulus processing is impaired in our group of patients.

An important limitation of this study was the inability to collect data from the cerebellum due to technical constraints. We tried to overcome this limitation as far as we could, by training the subjects outside the scanner so that they were familiar with the stimulus during FES conditions. However, the cerebellum is thought to be part of the motor control loop, and it has been shown to be differentially involved during FES supported and nonsupported by volitional contribution [7]. Moreover, computing predictions of sensory consequences is seen in the literature as a major role of the cerebellum (together with the parietal cortex) within the sensory-motor control loop [51]. Further, the focus of this work was comparing the patients who show carryover effect against those who do not after the identical treatment based on FES. In this view, a group of patients with no treatment was not included as this was beyond the purpose of the current study. The number of recruited patients was limited, but nevertheless the results reported are statistically robust and point towards a biological basis for the carryover. Further larger prospective studies are recommended to explore those aspects.

## 5. Conclusions

In conclusion, we suggest that the mechanism of action of FES carryover is based on movement prediction and sense of agency/body ownership. In other words, the ability of a patient to plan the movement and to perceive the stimulation as a part of his/her own control loop is important for the FES carryover effect to take place. Although we point to abnormal responses in SMA and AG as indicators that FES carryover effect is unlikely, it might be that in future a behavioural questionnaire devoted to the evaluation of self/non-self-perceived FES-induced movement might be useful in predicting the carryover effect in routine clinical settings.

## Figures and Tables

**Table 1:** Patients characteristics.

Pz	Age [years]	Sex [male/female]	Site of lesion	Type of stroke	Time [months]	Gait velocity [m/s]	Endurance velocity [m/s]	Paretic step length [mm]	TAAI [0–1]	MRC [1–5]	Capacity Score	Mirror movements [condition]	Training device	Carryover effect evaluation
pz01	53	M	L GP	H	37	0.43	1.02	490	0.68	3	20.67	V	WalkAid	—
pz02	23	M	R MCA	H	23	0.64	0.82	486	0.60	3	20.30	No	WalkAid	Yes
pz03	38	F	R GP	I	23	0.25	0.28	194	0.48	2	8.15	V	Bioness	No
pz04	64	F	L MCA	H + I	13	0.32	0.50	345	0.56	3	14.33	No	WalkAid	Yes
pz05	19	M	L MCA	H	44	0.82	1.01	561	0.44	3	23.42	No	WalkAid	No
pz06	47	F	L GP	H	44	0.52	0.82	513	0.46	3	21.26	FV, V	WalkAid	Yes
pz07	25	F	R MCA	I	30	0.70	0.98	591	0.62	3	24.50	FV, V	WalkAid	No
pz08	46	M	R GP	I	13	0.49	0.58	420	0.43	3	17.31	FV, V	WalkAid	No
pz09	57	M	L caudate nucleus	I	6	0.33	0.50	310	0.70	2	12.97	No	Bioness	—
pz10	49	M	R MCA	I	89	0.33	0.58	340	0.65	2	14.26	V	WalkAid	—
pz11	45	F	R MCA	I	9	0.26	0.71	250	0.68	3	11.18	No	Bioness	—
pz12	57	M	R parietal sub. + BG	I	18	0.39	0.75	350	0.68	2	14.93	No	WalkAid	No
pz13	33	M	L parac. lobule	I	6	0.34	0.32	410	0.82	3	16.25	No	WalkAid	No
pz14	61	F	R MCA	H	158	0.40	1.36	430	0.69	2	18.50	No	WalkAid	Yes

Minimum to maximum scores (or units) are expressed under each outcome score. Pz: patient; M: male; F: female; R: right; L: left; MCA: middle cerebral artery; ACA: anterior cerebral artery; GP: Globus Pallidus; parac.: paracentral; sub.: subcortical; BG: Basal Ganglia; H: haemorrhagic; I: ischemic; time: time since stroke at the time of first assessment; TAAI: Tibialis Anterior Activation Index; MRC: Medical Research Council index; FV: fMRI protocol condition, attempted voluntary movement with concurrent FES; V: fMRI protocol condition, voluntary movement without FES.

**Table 2:** Brain regions activation.

	MNI coordinates			*Z*	Side	ROI
	*x*	*y*	*z*			
(i) Patients group						
V > Rest	−2	−2	46	4.24^*∗*^	i	Cingulate g.
	2	−18	70	3.32^*∗*°^	i	SMA
	0	−16	70	3.31°	c/i	SMA
	−4	−24	74	3.31^*∗*^	c	Parac. lobule
	4	−20	62	2.98°	i	M1
FV > Rest	−6	−4	50	5.06^*∗*°^	c	SMA
	−14	4	24	3.88^*∗*^	c	Caudate nucleus
	−20	−36	70	3.34^*∗*^	c	Postcentral g.
	−4	−20	72	3.09^*∗*^	c	Parac. lobule
FP > Rest	24	18	36	3.81^*∗*^	i	Sup. front. g
	48	−66	40	3.29^*∗*^	i	AG
	52	−62	36	3.16°	i	AG
P > Rest	2	−52	32	4.12^*∗*^	i	Cingulate g.
	10	−55	22	3.40^*∗*^	i	Precuneus
	16	−26	52	3.66^*∗*^	i	SMA
	−10	−40	58	3.47^*∗*^	c	S1
	−6	−24	54	3.46^*∗*^	c	Parac. lobule
	−2	−30	54	3.28°	c	M1
	0	−28	54	3.23°	c/i	M1
INTER	0	−62	40	4.41^*∗*^	c/i	Precuneus
	−42	−64	40	3.30^*∗*^	c	AG
	−44	−58	36	3.20^*∗*^	c	AG
	10	−54	28	3.20^*∗*^	i	Precuneus

(ii) Conjunction between patients group						
and healthy control group						
V > Rest	2	4	46	4.62^*∗*^	i	SMA
	2	−16	66	4.07^*∗*°^	i	SMA
	4	−20	62	3.92°	i	M1
	0	−22	64	3.76°	c/i	M1
	0	−4	46	3.26^*∗*^	c/i	Cingulate g.
	0	−12	54	3.23°	c/i	SMA
FV > Rest	2	−14	64	4.36^*∗*°^	i	SMA
	6	−12	64	3.85°	i	PM
	6	−20	64	3.83°	i	M1
	−2	−20	64	3.80°	c	M1
	2	4	46	3.74^*∗*^	i	SMA
	−4	−6	50	3.69^*∗*°^	c	SMA
	−14	−36	66	3.56^*∗*^	c	Parac. lobule
	64	−22	28	3.30°	i	SII
FP > Rest	−2	−14	68	3.13^*∗*^	c	Parac. lobule
	−4	−24	62	3.11^*∗*°^	c	M1
P > Rest	−2	−30	54	3.78^*∗*°^	c	M1
	0	−28	56	3.73°	c/i	M1
	4	−24	58	3.38^*∗*°^	i	M1
	−10	−40	58	3.35^*∗*°^	c	S1
	−4	−22	68	3.31^*∗*^	c	Parac. lobule
INTER	0	−60	40	3.26^*∗*^	c/i	Precuneus

(ii) Patients group > healthy control group						
FV > Rest	−4	−44	22	3.73^*∗*^	c	Cingulate g.
	−28	−74	46	3.65^*∗*^	c	Supramarg. g.
	−8	−70	44	3.48^*∗*^	c	Precuneus
FP > Rest	26	18	40	4.50^*∗*^	i	Sup. front. g.
	10	−50	42	4.49^*∗*^	i	Precuneus
	2	−22	40	4.46^*∗*^	i	Cingulate g.
	−42	−52	46	4.15^*∗*^	c	Supramarg. g.
	50	−58	36	3.71^*∗*°^	i	AG
	28	34	34	3.61^*∗*^	i	Middle front. g.
	20	34	30	3.53^*∗*^	i	Sup. front. g.
	56	24	38	3.42^*∗*^	i	Inf. front. g.
	38	−64	44	3.41^*∗*^	i	AG
	−28	−58	58	3.34^*∗*^	c	Sup. parietal g.
	−46	−52	44	3.31^*∗*^	c	Supramarg. g.
P > Rest	6	−40	36	5.26^*∗*^	i	Cingulate g.
	−4	−32	30	4.68^*∗*^	c	Cingulate g.
	50	−66	36	3.94^*∗*°^	i	AG
	−34	−64	34	3.77°	c	AG
INTER	16	−54	26	3.60^*∗*^	i	Precuneus
	−50	18	36	3.52^*∗*^	c	Middle front. g.
	−40	−22	40	3.44^*∗*^	c	Postcentral g.
	−18	28	46	3.39^*∗*^	c	Sup. front. g.

(ii) Healthy control group > patients group						
FV > Rest	−32	−44	60	3.81^*∗*^	c	Sup. parietal g.

(iii) Responders > nonresponders						
V > Rest	−6	−4	50	3.62^*∗*°^	c	SMA
	−22	−38	66	3.62^*∗*^	c	Postcentral g.
	10	−20	64	3.15°	i	M1
FV > Rest	−4	−6	52	3.70^*∗*°^	c	SMA
FP > Rest	−2	−10	66	3.81^*∗*°^	c	SMA
	−34	48	14	3.36^*∗*^	c	Middle front. g.
P > Rest	46	−10	18	3.45^*∗*^	i	Rolandic operc.

	30	−42	50	3.74^*∗*^	i	Supramarg. g.

(iii) Nonresponders > responders						
INTER	−40	−62	36	4.32^*∗*°^	c	AG
	−20	−24	64	3.24^*∗*^	c	Precentral g.
	10	−16	68	3.47^*∗*^	i	SMA
	−36	0	48	4.53^*∗*^	c	Precentral g.
	22	6	46	3.69^*∗*^	i	Sup. front. g.
	−10	−2	52	3.38^*∗*^	c	SMA
	−16	−6	70	3.58^*∗*^	i	Sup. front. g.
	−32	−26	46	3.62^*∗*^	c	Postcentral g.

°Significant activation at Familywise Error (FWE) corrected *p* < 0.05 within predefined regions of interest. ^*∗*^Significant activation at uncorrected *p* < 0.001. ROI: region of interest; FV: fMRI protocol condition, attempted voluntary movement with concurrent FES; V: fMRI protocol condition, voluntary movement without FES; FP: fMRI protocol condition, FES-induced movement, with no attempt to move the ankle; P: fMRI protocol condition, passive movement (by the experimenter) of the subject's ankle without FES; c: contralateral side; i: ipsilateral side; g.: gyrus; parac.: paracentral; sup.: superior; front.: frontal; supramarg.: supramarginal; inf.: inferior; operc.: operculum; SMA: supplementary motor area; M1: primary motor cortex; AG: angular gyrus; S1: primary somatosensory cortex; PM: premotor cortex; SII: secondary somatosensory cortex.
